# Challenges Encountered during the Treatment of Acute Mesenteric Ischemia

**DOI:** 10.1155/2020/5316849

**Published:** 2020-03-31

**Authors:** Mateusz Jagielski, Jacek Piątkowski, Marek Jackowski

**Affiliations:** Department of General, Gastroenterological and Oncological Surgery, Collegium Medicum Nicolaus Copernicus University, Toruń, Poland

## Abstract

**Results:**

Acute ischemia of the bowel mesentery was diagnosed in 41 patients (27 women and 14 men; mean age, 65.4 years). All patients underwent laparotomy. For 13 (31.71%) patients, surgery was performed within the first 24 hours of the clinical symptom onset. Mesenteric artery embolectomy without intestine resection was performed for 7 (17.07%) patients. Partial intestine resection due to necrosis was performed for 21 (51.22%) patients. Exploratory laparotomy without a therapeutic procedure was performed for 13 (31.71%) patients. Fifteen (36.59%) patients were discharged home in good general condition. Twenty-six (63.41%) patients died. The time from the clinical symptom onset until intervention exceeded 24 hours for all patients who died. Surgery within the first 24 hours reduced mortality associated with acute mesenteric ischemia (*P* = 0.001). Female sex, age older than 65 years, obesity (body mass index > 30), diabetes, chronic kidney disease, and smoking were adverse prognostic factors for increased mortality for patients with acute bowel ischemia.

**Conclusion:**

The time from clinical symptoms to acute mesenteric ischemia treatment was the main prognostic factor and helped determine appropriate management. Early diagnosis and rapid intervention improved treatment outcomes and survival.

## 1. Introduction

Acute mesenteric ischemia is a rare disorder defined as the sudden disruption of the blood supply to the intestine [1–4]. If left untreated, it will lead to complications and irreversible consequences such as necrosis of the intestinal wall and death [1–4]. We distinguished occlusive and nonocclusive types of acute ischemia of the bowel mesentery based on their cause [1–3]. Occlusive causes of acute mesenteric ischemia include mesenteric artery embolism (50% of cases), mesenteric artery thrombosis (15-25%), and celiac vein thrombosis (5-15%) [3, 4]. Diverse causes and nonspecific clinical symptoms of patients with acute mesenteric ischemia hinder the diagnostic process, often resulting in delayed diagnosis and late therapeutic intervention.

Despite numerous publications describing this problem, acute mesenteric ischemia is a great clinical challenge [1–6]. Most publications in the available literature regarding the management of acute mesenteric ischemia are reviews [1–6] and retrospective case series reports [7–10]. There are no clear guidelines for the therapeutic management of these patients. Therefore, further studies of acute bowel ischemia focused on creating uniform diagnostic and therapeutic standards are necessary [1–10].

This work is aimed at presenting guidelines for the management of acute mesenteric ischemia based on an analysis of a variety of treatments and identifying prognostic factors that can be used to determine therapeutic options to improve treatment outcomes and facilitate optimal decision-making during daily clinical practice.

## 2. Materials and Methods

We performed a retrospective analysis of all patients diagnosed with acute mesenteric ischemia of occlusive cause who were hospitalized between January 1, 2002, and December 31, 2018, at our institution. Patients with symptoms of chronic mesenteric ischemia and patients with acute bowel ischemia caused by nonocclusive factors were excluded from the study.

**Scheme 1:**
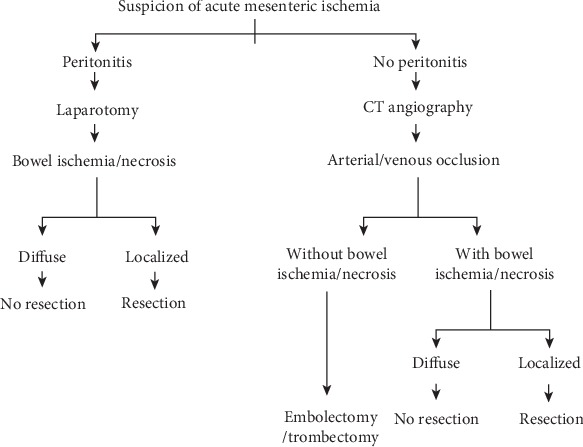
Diagnostic and treatment algorithm in patients with suspected acute mesenteric ischemia.

**Figure 1:**
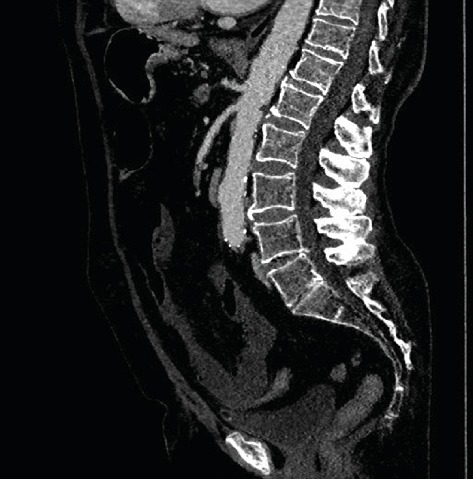
Computed tomographic angiography (angio-CT) of abdominal blood vessels in a patient with acute mesenteric ischemia of embolic cause. A clot obstructing the vessel lumen is visible.

Therapeutic management dependent on clinical signs and the extent and type of pathology identified are described in Scheme 1. All patients with symptoms of peritonitis were referred for urgent laparotomy, and acute mesenteric ischemia was diagnosed intraoperatively. For patients without symptoms of peritonitis, acute mesenteric ischemia was diagnosed preoperatively based on computed tomographic angiography (angiocomputed tomography (angio-CT)) of the abdominal vasculature (Figure 1). In this group of patients, acute mesenteric ischemia was confirmed intraoperatively.

We analyzed patient demographics, clinical symptoms, concomitant diseases, blood test results, and data regarding the type and extent of gastrointestinal lesions, type of occlusive cause of the condition, and type of surgical intervention. The study group was divided into subgroups based on the cause of ischemia, type of therapeutic intervention, and survival for the purpose of statistical analyses.

The Portsmouth modification of the Physiologic and Operative Severity Scoring System for Enumeration of Morbidity and Mortality (P-POSSUM) was used to assess all patients. Moreover, we analyzed therapeutic management strategies during the postoperative period and short-term and long-term treatment outcomes during follow-up. Prognostic factors associated with survival were subject to analysis. In the event of postoperative death, the exact cause of death was identified based on a postmortem examination or the diagnosis noted on the death certificate when autopsy was not performed. Factors associated with mortality were analyzed based on the intervention.

All statistical analyses were performed using R package v. 3.2.3. Quantitative variables are shown as medians and interquartile ranges. Qualitative data are presented as numbers and percentages. Differences between qualitative variables were assessed using the chi-square test or Fisher's exact test. Statistical significance of the effect of a quantitative variable for two independent groups was verified using the Mann-Whitney test, whereas the Kruskal-Wallis test was used for three groups. A stepwise logistic regression analysis was performed. Two-tailed tests were performed, and *P* ≤ 0.05 was considered significant.

## 3. Results

Forty-one patients (27 women and 14 men; mean age, 65.4 (range, 35-88) years) with acute mesenteric ischemia were hospitalized between January 1, 2002, and December 31, 2018. Occlusive causes of acute mesenteric ischemia were defined as either embolic (25/41 patients (60.98%)) or thrombotic (16/41; 39.02%). Arterial embolism was predominant among the thromboembolic causes (14/41; 34.15%). Venous thrombosis was diagnosed in 2 of 41 (4.87%) patients.

Arterial embolism most often involved the superior mesenteric artery (19/25; 76%). Less often, it involved the inferior mesenteric artery (4/25; 16%) or both the superior and inferior mesenteric arteries (2/25; 8%). A blood clot affected the upper mesenteric artery most often (10/14; 71.42%). Less often, it affected the lower mesenteric artery (2/14; 14.29%) or both mesenteric arteries (2/14; 14.29%). The two patients with venous thrombosis experienced a blood clot in the upper mesenteric vein.

Patients with embolic causes were younger (64 vs. 78.5 years; *P* < 0.001) and experienced atrial fibrillation more often (92% vs. 6.2%; *P* < 0.001) compared to patients with thrombosis. The majority of patients (92%) with atrial fibrillation were using anticoagulants; only 2 (8%) patients did not use anticoagulant therapy. Thrombosis was more common in patients with concomitant atherosclerosis (56.2% vs. 0%; *P* < 0.001) and hypercholesterolemia (81.2% vs. 40%; *P* = 0.009). There were no statistically significant differences regarding the type of clinical symptoms and results of laboratory blood tests based on the cause of acute mesenteric ischemia.  Table 1 presents a comparison of patients with different occlusive causes of acute mesenteric ischemia.

Abdominal pain was the most common clinical symptom of acute mesenteric ischemia, occurring in 38 (92.68%) patients. Symptoms of acute peritonitis were noted in 29 (70.73%) patients. Twenty-five (60.98%) patients were diagnosed with de novo multiorgan failure (most often acute renal failure and cardiovascular failure). Angio-CT of the abdominal vessels was performed for 13 (31.71%) patients.  Table 2 presents the clinical signs observed in patients with acute mesenteric ischemia.

The average time from clinical symptom onset to surgical intervention was 43.525 (range, 8-120) hours. All patients underwent laparotomy. Changes typical for ischemia and necrosis were found in 34 (82.93%) patients. Surgery was performed within the first 24 hours from the clinical symptom onset for 13 (31.71%) patients; of these patients, 7 (17.07%) underwent surgical intervention performed within 12 hours of the symptom onset. All seven patients underwent embolectomy of the mesenteric artery without resection due to the lack of ischemia or necrosis of the intestine. Based on an analysis of the duration of clinical symptoms, extent of gastrointestinal tract involvement, and type of intervention, the first 12 hours from the clinical symptom onset is the optimal time for intervention. During that time, it is possible to perform vascular surgery effectively while avoiding resection of the intestine (*P* < 0.001).

**Figure 2:**
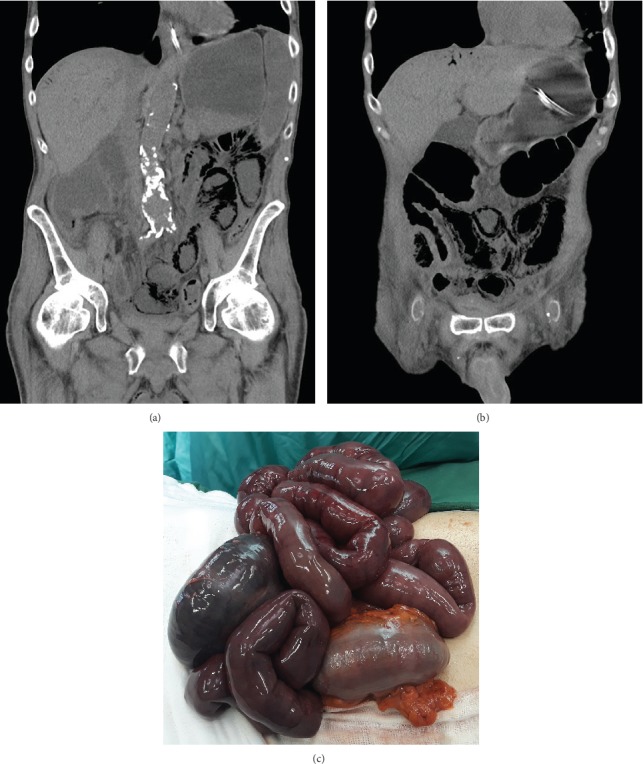
(a, b) Multiphase computed tomographic angiography (angio-CT) of the abdominal cavity and pelvis in a patient with acute mesenteric ischemia without symptoms of peritonitis. Signs of intestinal obstruction and pneumatosis of the small and large intestinal walls due to an embolism of the superior and inferior mesenteric arteries. (c) Intraoperative view of the same patient. Widespread necrosis of the walls of the small intestine and colon was revealed during exploratory laparotomy. Resection was not performed due to the advancement and extent of necrotic changes.

Partial resection of the intestine due to necrotic changes was performed for 21 (51.22%) patients: 5 underwent resection of the small intestine (anastomosis was performed for 3 patients; 2 patients had a stoma); 6 patients underwent resection of the colon with stoma formation; and 10 patients underwent resection of the small intestine and the right side of the colon (anastomosis was performed for 3 patients; 7 patients had a stoma). In 3/21 patients, a vascular procedure (embolectomy of the superior mesenteric artery) combined with bowel resection was performed. Exploratory laparotomy without a therapeutic procedure (Figures 2(a)–2(c)) was performed for 13 (31.71%) patients; resection was not performed due to the extent and advancement of lesions within the small and large intestines.

There were no statistically significant differences in demographics, comorbidities, clinical symptoms, laboratory results, organ failure, or mortality based on the extent of necrosis for a group of 21 patients who underwent resection of the intestine.

During the postoperative period, 30 (73.17%) patients required hospitalization in the intensive care unit due to continuing signs of organ failure. The average hospital stay was 3.03 (range, 1-12) days. Twenty-nine (70.73%) patients required inotropes during the postoperative period. Thirty-four (82.93%) patients required intravenous antibiotic therapy. Eleven (26.83%) patients required total parenteral nutrition. The impact of the final treatment and its outcomes are represented in Table 3.

During the postoperative period, six (14.63%) patients underwent repeat surgery: three patients underwent resection of an intestinal fragment; two patients underwent reoperation due to an intestinal anastomosis leak; and one patient underwent reoperation due to a peritoneal hematoma.

Twenty-six (63.41%) patients died (21 women and 5 men; *P* = 0.008; mean age, 79.18 (range, 37-88) years; *P* = 0.003).The most common cause of death was multiorgan failure (15/26; 57.69%), followed by myocardial infarction (6/26; 23.08%) and sepsis (3/26; 11.54%). The cause of death was not established for 2 of these 26 (7.69%) patients. For all patients who died during intervention, the time from clinical symptom onset to intervention exceeded 24 hours. No deaths occurred among patients who underwent surgical intervention within 24 hours of the symptom onset. Therefore, performing surgery within the first 24 hours of illness reduced the mortality of patients with acute mesenteric ischemia (*P* = 0.001).

Fifteen (36.59%) patients were discharged home in good general condition. The mean follow-up time after discharge was 484 (range, 7-1084) days. Acute bowel ischemia did not recur during the observation period of this group of patients. Two of 15 (13.33%) patients were diagnosed with short bowel syndrome, which required long-term parenteral nutrition.

Factors associated with the survival of patients with acute mesenteric ischemia and laboratory test results (Tables 3 and 4, respectively) were used to create a logistic regression model to determine risk factors for perioperative mortality. Female sex, age older than 65 years, obesity (body mass index > 30), diabetes, chronic kidney disease, and smoking are adverse prognostic factors associated with increased mortality. Other adverse prognostic factors include fever, abnormal blood test results (elevated inflammatory markers, leukocytosis, thrombocytosis, C-reactive protein, and procalcitonin), increased factors for kidney injury (increased creatinine and hyperkalemia), and lactic acidosis. Multiorgan failure before surgery, needing inotropic agents, and needing intravenous antibiotics during the perioperative period are also predictors of poor prognoses for patients with acute mesenteric ischemia.

## 4. Discussion

Acute mesenteric ischemia is a rare disorder that accounts for approximately 0.09% to 0.2% of all acute surgical admissions [5, 11–13]. The majority of patients with acute bowel ischemia are elderly and have numerous comorbidities that are considered adverse prognostic factors [14, 15]. Nevertheless, neither age nor comorbidities should be considered contraindications for radical surgical management of acute mesenteric ischemia. The decision to withdraw treatment should be based on validated prognostic scales that are used to objectively assess the general condition based on physiological variables and factors associated with greater perioperative mortality. Most reports have suggested using the P-POSSUM scale to accurately assess the risk of complications and death during the perioperative period and during vascular surgery [13, 16, 17]. Our study showed that the scores of the P-POSSUM scale corresponded closely with the actual treatment outcomes of patients with acute mesenteric ischemia. The P-POSSUM scale can be used as a guide when determining the most appropriate treatment or whether to withdraw definitive treatment.

Undoubtedly, diagnostic and therapeutic procedures for patients with suspected acute mesenteric ischemia should be performed urgently [5–7]. The differential diagnosis of acute mesenteric ischemia is a prognostic factor for disease progression. Delaying surgical treatment is closely associated with increased mortality [8–10, 15]. Acceptable treatment results and reduced mortality are only possible during early-stage disease (0-24 hours from symptom onset) for patients with acute mesenteric ischemia. Only an early diagnosis and rapid intervention involving restoration of the blood supply to the intestine significantly improved the results of acute mesenteric ischemia treatment and led to reduced complications and mortality associated with this illness. Poor treatment outcomes often resulted from delayed diagnosis and, thus, delayed therapeutic intervention in this group of patients. Results of other studies available in literature support these results [8–10, 15].

The time from symptom onset to treatment commencement not only affects treatment outcomes but also helps to determine the appropriate treatment method. Immediate restoration of the blood supply of the viscera [5–10] is the priority for acute mesenteric ischemia treatment. The optimal time for intervention is during the first 12 hours from the clinical symptom onset, when it is possible to perform vascular surgery effectively without requiring intestinal resection. During the early stages of disease (up to 12 hours after symptom onset), embolectomy or thrombectomy is the definitive treatment method for preventing disease progression. Despite intravascular treatment, most patients require exploratory laparotomy to assess intestinal viability, which often alleviates the need for revision surgery during the recovery period. When surgery is performed more than 24 hours after the symptom onset, it is necessary to perform a partial resection of the intestine affected by necrosis [8–10]. Despite these results, the period between 12 and 24 hours from symptom onset remains controversial. During this time, vascular surgery can be performed without resection and patients can be carefully observed. Repeat laparotomy is a possibility if signs of necrosis appear. It is also possible to perform resection during the first laparotomy, thus preventing further procedures. However, disease management also continues to be controversial.

The effectiveness of other intervention methods for acute ischemia has been demonstrated in the field of vascular surgery [5, 6, 18–20]. In our opinion, intravascular intervention is the only method of acute bowel ischemia treatment that is controversial because intestinal viability assessment through direct inspection, such as during laparotomy, is absolutely crucial. Intravascular intervention may be a therapeutic alternative for patients with early-stage illness or for patients at high surgical risk according to the P-POSSUM scale but without the characteristics of peritonitis on physical examination. Currently, only those two groups of patients could potentially benefit from minimally invasive methods of vascular surgery. At our center, such a treatment method has not been implemented despite the availability of vascular surgeons and facilities. In the near future, perhaps with the development of new treatments, it may be possible to implement endovascular interventions for patients with acute mesenteric ischemia during the early stage of illness, when the risk of bowel necrosis is very small, or as hybrid therapy combined with exploratory laparoscopy for assessing intra-abdominal ischemia.

Angio-CT of the abdominal cavity is the gold standard for diagnosing acute mesenteric ischemia [5, 6, 12, 19]. There is no rationale for performing angio-CT in the presence of peritonitis symptoms. In contrast to the results of other publications available in the literature [9], our study did not indicate that angio-CT improves treatment outcomes or survival of patients with acute mesenteric ischemia. Moreover, it can sometimes delay the diagnosis and implementation of definitive treatment. Therefore, it seems reasonable to suggest diagnostic and therapeutic management guidelines including angio-CT for suspected acute mesenteric ischemia without clinical signs of peritonitis.

No single laboratory parameter specific for acute mesenteric ischemia has been identified to date [7–10, 15]. Increased inflammatory markers and lactic acidosis, which are adverse prognostic factors, are not specific for acute mesenteric ischemia and confirm ongoing peritoneal inflammation and intestinal necrosis. The aforementioned factors as well as increased factors associated with renal failure can only be interpreted in the context of the overall clinical picture. Due to rapid disease progression, immediate definitive treatment is crucial when acute mesenteric ischemia is strongly suspected regardless of the results of additional diagnostic tests [5, 6, 12].

Our results regarding the poor prognosis of patients with organ failure requiring intravenous inotropes or antibiotics should be viewed in a similar context. Poor survival of this group of patients is usually associated with the advancement of gastrointestinal tract necrosis that requires intensification of conservative treatment.

The main limitations of our study were its lack of randomization, retrospective nature, and the fact that it involved a specific group of patients from a single center. However, due to the low incidence and clinical course of acute mesenteric ischemia, it is not possible to plan and conduct randomized clinical trials involving this group of patients. Most of the available information regarding the diagnostic and therapeutic management of patients with acute mesenteric ischemia has been obtained from retrospective studies, such as ours, thus justifying the need for and rationale behind such publications.

## 5. Conclusion

This study suggests diagnostic and therapeutic management for patients with acute mesenteric ischemia based on various treatment strategies. Suspicion of acute mesenteric ischemia is a decisive prognostic factor for disease progression. When commencing treatment, one should remember that immediate restoration of the blood supply to the viscera is a priority. The time from the clinical symptom onset to the initiation of definitive treatment for acute mesenteric ischemia is the main prognostic factor and helps to determine the appropriate treatment method. Only an early diagnosis and rapid intervention can improve treatment outcomes and survival of patients with acute mesenteric ischemia.

## Figures and Tables

**Table 1:** Comparison between groups according to the etiology of acute mesenteric ischemia.

Factors	Mesenteric thrombosis (*N* = 16)	Mesenteric embolism (*N* = 25)	*P* value
Demographic information			
Gender			0.323
Female	12 (75.0%)	15 (60.0%)	
Male	4 (25.0%)	10 (40.0%)	
Age			0.027
Median (Q1, Q3)	78.500 (71.250, 82.250)	64.000 (58.000, 80.000)	
Range	58.000-88.000	35.000-85.000	
Comorbid medical condition			
Obesity	2 (12.5%)	9 (36.0%)	0.098
Coronary artery disease	8 (50.0%)	14 (56.0%)	0.707
Congestive heart failure	8 (50.0%)	13 (52.0%)	0.901
Atrial fibrillation	1 (6.2%)	23 (92.0%)	<0.001
Hypertension	15 (93.8%)	17 (68.0%)	0.052
Hypercholesterolemia	13 (81.2%)	10 (40.0%)	0.009
Diabetes	8 (50.0%)	9 (36.0%)	0.375
Tobacco abuse	2 (12.5%)	9 (36.0%)	0.098
Chronic obstructive pulmonary disease	1 (6.2%)	4 (16.0%)	0.352
Chronic renal failure	7 (43.8%)	8 (32.0%)	0.446
Atherosclerotic disease	9 (56.2%)	0 (0.0%)	<0.001
Results of treatment			
Risk of morbidity (P-POSSUM)			0.017
Median (Q1, Q3)	93.000 (81.500, 96.500)	70.000 (41.000, 88.000)	
Range	31.000-100.000	25.000-100.000	
Risk of mortality (P-POSSUM)			0.029
Median (Q1, Q3)	32.500 (17.750, 54.250)	10.000 (3.000, 26.000)	
Range	2.000-85.000	1.000-86.000	
Bowel resection	12 (75%)	9 (36.0%)	0.041
Vascular procedure	0 (0.0%)	7 (28.0%)	0.020
Alive	5 (31.2%)	10 (40.0%)	0.570
Dead	11 (68.8%)	15 (60.0%)	0.570

**Table 2:** Presenting symptoms in patients with acute mesenteric ischemia.

Symptoms	*n* (%)
Abdominal pain	38 (92.7%)
Peritonitis	29 (70.7%)
Fever	20 (48.8%)
Diarrhea	13 (31.7%)
Vomiting	13 (31.7%)
Evidence of shock	8 (19.5%)
Symptoms of bleeding into lumen of gastrointestinal tract	7 (17.1%)

**Table 3:** Factors effecting mortality in the patients with acute mesenteric ischemia.

Factors	Alive (*N* = 15)	Dead (*N* = 26)	*P* value
*Gender*			0.008
Female	6 (40.0%)	21 (80.8%)	
Male	9 (60.0%)	5 (19.2%)	
*Age*			0.003
Median (Q1, Q3)	61.000 (54.500, 72.000)	79.000 (67.250, 82.750)	
Range	35.000-80.000	37.000-88.000	
*Amount of comorbid medical condition*			0.989
Median (Q1, Q3)	5.000 (4.000, 6.000)	5.000 (3.250, 6.750)	
Range	2.000-7.000	0.000-9.000	
*Etiology of acute mesenteric ischemia*			0.570
Thrombosis	5 (33.3%)	11 (42.3%)	
Embolism	10 (66.7%)	15 (57.7%)	
*Type of vessel*			0.427
Artery	14 (93.3%)	25 (96.2%)	
Vein	1 (6.7%)	1 (3.8%)	
*Organ failure before intervention*			0.003
Absent	10 (66.7%)	3 (11.5%)	
Present	5 (33.3%)	23 (88.5%)	
*CT angiography before intervention*			0.091
Absent	8 (53.3%)	20 (76.9%)	
Present	7 (46.7%)	6 (23.1%)	
*Risk of morbidity (P-POSSUM)*			<0.001
Median (Q1, Q3)	41.000 (35.500, 74.000)	92.500 (81.750, 97.000)	
Range	25.000-96.000	43.000-100.000	
*Risk of mortality (P-POSSUM)*			<0.001
Median (Q1, Q3)	6.000 (2.000, 10.000)	34.000 (18.500, 55.000)	
Range	1.000-34.000	3.000-86.000	
Bowel resection	8 (53.3%)	13 (50.0%)	0.275
Vascular procedure	7 (46.7%)	0 (0.0%)	<0.001
Antibiotics	9 (60.0%)	25 (96.2%)	0.003
Total parenteral nutrition	6 (40.0%)	5 (19.2%)	0.148
Inotropes	5 (33.3%)	24 (92.3%)	<0.001

**(a):** 

Parameter in blood test	Alive (*N* = 15)	Dead (*N* = 26)	*P* value
*Hemoglobin (g/dl)*			0.146
Median (Q1, Q3)	14.300 (12.750, 15.300)	13.000 (11.800, 13.500)	
Range	9.800-17.700	9.600-19.400	
*Leukocytes (mm^3^)*			0.009
Median (Q1, Q3)	15.090 (13.000, 18.035)	20.500 (16.700, 26.710)	
Range	8.100-33.010	9.700-35.800	
*Thrombocytes (mm^3^)*			0.011
Median (Q1, Q3)	263.000 (204.500, 401.500)	489.000 (323.000, 503.000)	
Range	154.000-505.000	110.000-555.000	
*C-reactive protein (mg/L)*			<0.001
Median (Q1, Q3)	111.400 (32.725, 210.400)	302.110 (224.500, 340.145)	
Range	27.700-404.600	43.700-585.700	
*Procalcitonin (μg/l)*			<0.001
Median (Q1, Q3)	1.180 (0.235, 2.125)	11.450 (7.797, 12.850)	
Range	0.050-9.500	1.100-26.600	
*Creatinine (mg/dl)*			<0.001
Median (Q1, Q3)	0.970 (0.880, 1.520)	1.770 (1.450, 2.200)	
Range	0.650-2.150	0.430-2.560	
*BUN (mg/dl)*			0.070
Median (Q1, Q3)	19.600 (16.675, 46.275)	58.250 (35.675, 67.900)	
Range	15.500-78.500	15.200-80.000	
*pH*			<0.001
Median (Q1, Q3)	7.385 (7.333, 7.408)	7.210 (7.140, 7.250)	
Range	7.200-7.440	7.080-7.330	
*Lactate (mmol/l)*			<0.001
Median (Q1, Q3)	2.700 (1.950, 5.400)	7.050 (5.890, 7.800)	
Range	1.100-7.000	4.000-9.500	
*Sodium (mEq/l)*			0.922
Median (Q1, Q3)	139.000 (136.500, 142.000)	140.000 (135.000, 143.000)	
Range	128.000-154.000	130.000-151.000	

**(b):** 

Parameter in blood test	Alive (*N* = 15)	Death (*N* = 26)	*P* value
*Potassium (mEq/l)*			0.031
Median (Q1, Q3)	4.100 (3.790, 4.550)	5.300 (4.500, 5.690)	
Range	3.100-6.010	3.240-6.210	
*D-dimer (mg/l)*			0.732
Median (Q1, Q3)	4.280 (3.897, 4.612)	3.760 (3.130, 5.250)	
Range	2.390-6.600	2.500-5.550	
*INR*			0.164
Median (Q1, Q3)	1.100 (1.052, 1.133)	1.200 (1.070, 1.415)	
Range	0.900-1.230	0.890-1.800	
*Troponin T (ng*/*ml)*			0.356
Median (Q1, Q3)	0.440 (0.351, 0.540)	0.565 (0.351, 0.835)	
Range	0.245-0.930	0.321-0.894	
*CK-MB (μg/l)*			0.845
Median (Q1, Q3)	7.200 (7.050, 8.700)	7.420 (7.053, 7.850)	
Range	6.200-10.000	6.600-9.000	
*ALT (U/l)*			0.409
Median (Q1, Q3)	70.500 (57.250, 84.000)	89.500 (48.500, 103.750)	
Range	25.000-100.000	10.000-345.000	
*AST (U/l)*			0.090
Median (Q1, Q3)	69.500 (44.750, 78.500)	85.500 (50.750, 110.500)	
Range	28.000-105.000	16.000-456.000	
*Amylase (U/l)*			0.251
Median (Q1, Q3)	104.000 (97.000, 148.250)	150.000 (101.500, 221.500)	
Range	34.000-403.000	41.000-511.000	

## Data Availability

The database with detailed data on patients data used to support the findings of this study are available from the corresponding author upon request. Data are available from corresponding author for researchers who meet the criteria for access to confidential data.
